# The prion protein family: a view from the placenta

**DOI:** 10.3389/fcell.2014.00035

**Published:** 2014-08-08

**Authors:** Samira Makzhami, Bruno Passet, Sophie Halliez, Johan Castille, Katayoun Moazami-Goudarzi, Amandine Duchesne, Marthe Vilotte, Hubert Laude, Sophie Mouillet-Richard, Vincent Béringue, Daniel Vaiman, Jean-Luc Vilotte

**Affiliations:** ^1^INRA, UMR1313 Génétique Animale et Biologie IntégrativeJouy-en-Josas, France; ^2^INRA, U892 Virologie et Immunologie MoléculairesJouy-en-Josas, France; ^3^INSERM, UMR-S1124 Signalisation et Physiopathologie Neurologique, Université Paris DescartesParis, France; ^4^Faculté Paris Descartes, UMR8104 CNRS, U1016 INSERM, Institut CochinParis, France

**Keywords:** prion, shadoo, doppel, placenta, development, mammals

## Abstract

Based on its developmental pattern of expression, early studies suggested the implication of the mammalian Prion protein PrP, a glycosylphosphatidylinositol-anchored ubiquitously expressed and evolutionary conserved glycoprotein encoded by the *Prnp* gene, in early embryogenesis. However, gene invalidation in several species did not result in obvious developmental abnormalities and it was only recently that it was associated in mice with intra-uterine growth retardation and placental dysfunction. A proposed explanation for this lack of easily detectable developmental-related phenotype is the existence in the genome of one or more gene (s) able to compensate for the absence of PrP. Indeed, two other members of the *Prnp* gene family have been recently described, Doppel and Shadoo, and the consequences of their invalidation alongside that of PrP tested in mice. No embryonic defect was observed in mice depleted for Doppel and PrP. Interestingly, the co-invalidation of PrP and Shadoo in two independent studies led to apparently conflicting observations, with no apparent consequences in one report and the observation of a developmental defect of the ectoplacental cone that leads to early embryonic lethality in the other. This short review aims at summarizing these recent, apparently conflicting data highlighting the related biological questions and associated implications in terms of animal and human health.

## Introduction

Studies on the neuropathology of transmissible spongiform encephalopathy (TSE) revealed that misfolded, aggregated conformers of the host-encoded cellular protein PrP are the major if not the sole constituent of the infectious agent termed prion (Prusiner, 1982). Prion pathologies affect humans, with the Creutzfeldt-Jakob and Kuru diseases for example, and animals, with for instance the bovine spongiform encephalopathy and sheep scrapie. PrP is a glycosylphosphatidylinositol-anchored, ubiquitously expressed, glycoprotein encoded by the *Prnp* gene. The PrP primary and tertiary structures are well conserved among mammals. Although many studies focused on PrP potential role in the central nervous system in association with prion neurotoxicity, the evolutionary conservation of this protein and the *Prnp* gene expression pattern suggested that PrP may exert important biological roles. Numerous functions were attributed to PrP such as its implication in various signal transductions, cell adhesion, neuroprotection, basic biology of embryonic and tissue-specific stem cells, T-cell regulation and immune function, oxidative stress homeostasis and synaptic function (Westergard et al., 2007; Linden et al., 2008; Zomosa-Signoret et al., 2008; Haigh et al., 2010; Resenberger et al., 2011; Schneider et al., 2011; Lopes and Santos, 2012, for recent reviews as well as associated reviews from this special issue). However, the precise PrP biological role remains rather elusive. It may relate to the multiplicity of partners with which PrP appears to interact, with noticeable differences according to the cell type considered and/or the physiological condition assessed. These diverse cellular proteins include transmembrane receptors, metal ion transporters, signaling molecules, cytoskeleton-associated proteins which could suggest that PrP is a key component of a versatile signaling scaffold complex that allows the activation of various biological pathways.

**Figure 1:**
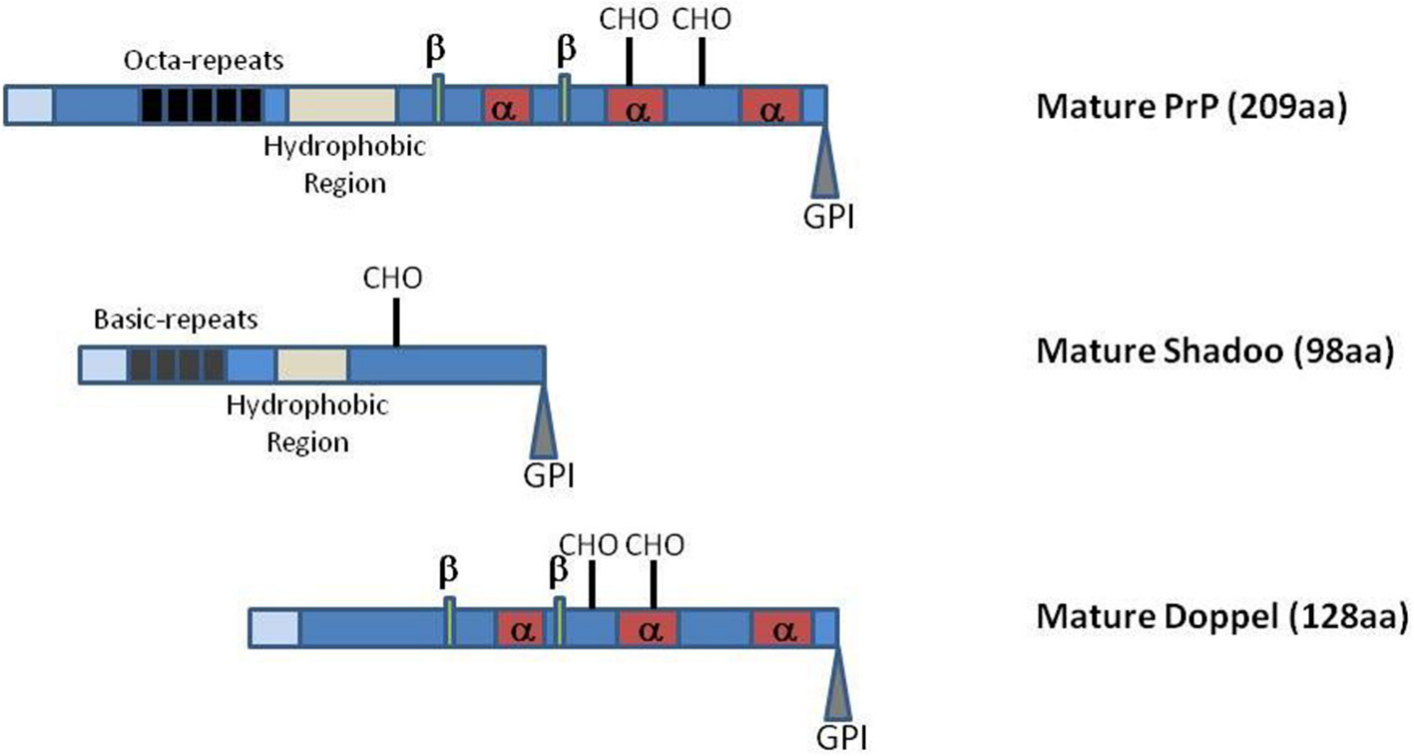
**Schematic representation of PrP, Shadoo and Doppel mature proteins**. Representations are not at scale. Light blue boxes: N-terminal charged regions of the mature proteins. α, α-helices; β, β-strands; CHO, N-glycosylation sites. GPI glycosylphosphatidylinositol. The PrP octa-repeat region and the Shadoo basic repeat region are indicated as well as the two protein hydrophobic domains.

One further difficulty in understanding PrP physiological function relates to the lack of obvious phenotype in *Prnp*-knockout mice, apart from resistance to TSE and the disappearance of the neurotoxicity associated with prions (Büeler et al., 1992, 1993; Manson et al., 1994). This absence of a strong phenotype could have various, non-mutually exclusive, origins such as (i) the lack of a crucial function of this protein in non-challenging breeding conditions, (ii) genetic adaptation of the animals to the lack of this protein, (iii) biological functional redundancy with other host-encoded proteins able to take over at least some of the PrP roles, and (iv) genetic robustness with PrP being involved in biological functions for which several pathways naturally exist. As mentioned above, the evolutionary conservation of the PrP protein during evolution, at least in mammals, and its involvement in the homeostasis of various stem cells appear somehow contradictory with an absence of important function(s). However, such function could be crucial only to allow adaptation of the animal against stressful conditions, situations that have not been yet really tested, or only in a few occasions, for *Prnp*-knockout mice. The second hypothesis has been challenged by the observation that invalidation only in adult neurons of the *Prnp* locus did not induce histopathological changes but resulted in a modulation of neuronal excitability (Mallucci et al., 2002), with very limited associated transcriptional alteration at a global brain level (Chadi et al., 2010). However, despite its high level of expression in the adult nervous system, PrP biological role might be crucial at earlier developmental stages in this and/or other tissues of the animal. Two other members of the prion gene family, that share with PrP some structural features (Figure 1), were recently described in the mammalian genomes, *Prnd* which encodes Doppel and *Sprn* which encodes Shadoo. These three genes are supposedly evolutionary derived from the retro-insertion event of a gene belonging to one of the four subfamilies of the Zinc transporter containing protein-encoding genes, the ZIP LIV-1 branch (Schmitt-Ulms et al., 2009; Ehsani et al., 2011). The existence of these two related proteins could be seen as being in favor of the existence of a biological redundant mechanism. Investigations toward testing this hypothesis led to apparently contradictory observations that have yet to be conciliated (Young et al., 2009; Daude et al., 2012; Passet et al., 2012).

Besides the understanding of the precise mechanism leading to the lack of strong phenotypes in *Prnp*-knockout mammals, a related and yet unsolved question is the tissue where and the developmental stage at which this phenotype would be the more likely to occur. Because of its involvement in TSE and of its higher level of expression in the adult nervous system, many studies first focused on this tissue. However, accumulating evidences associate PrP with stem cell biology and early developmental and regenerative processes. Of specific interest is thus the potential involvement of these proteins in the regulation of the extra-embryonic annexes and more precisely the placenta, based on the observation of the developmental regulation of this gene family in such normal or pathological tissues and on the association of alleles, including experimental gene knockouts, with phenotypes involving or at least recalling placental functional failures. This review summarizes our current knowledge on this specific topic.

## Prion protein gene family expression in extra-embryonic tissues

Available data on the regulation of the prion protein gene family in extra-embryonic tissues are limited and mostly related to the *Prnp* gene. Both *in situ* hybridization (Manson et al., 1992; Alfaidy et al., 2013) and uses of a *Prnp*-promoter/LacZ reporter transgene (Tremblay et al., 2007) allowed to identify mouse *Prnp*-gene expression in extra-embryonic membranes as early as 6.5 days post-coïtum, E6.5, the earliest analyzed developmental stage in these studies. This stage corresponds to a transition toward oxidative metabolism and is associated with an increase of the *Prnp* gene expression level (Miele et al., 2003). A similar early extra-embryonic expression of *Prnp* was reported in human (Donadio et al., 2007), rat (Tanji et al., 1995), and ruminant (Kubosaki et al., 2000; O'Rourke et al., 2011) placentas. However, this gene developmental regulation might differ between species, as observed for example in human where the high level of *Prnp* expression was restricted to the first trimester of pregnancy.

Transcription of members of the prion protein gene family was also detected at earlier developmental stages (Table 1). Mouse embryonic stem cells were reported to express the three genes (Miranda et al., 2011) and human embryonic stem cells to express at least the *Prnp* one (Krejciova et al., 2011; Lee and Baskakov, 2013). *Prnp* gene expression was also observed in human trophoblast cells (Alfaidy et al., 2013) and that of *Sprn* indirectly suspected to occur in mouse trophectoderm (Passet et al., 2012). We have detected *Sprn* and *Prnd* gene expressions in extra-embryonic tissues of mouse embryos at E10.5 and E13.5 (our unpublished observation and Young et al., 2011). In sheep, *Prnp* transcripts were also detected in mature and immature oocytes and at the morula stage (Thumdee et al., 2007) while in mouse, the recent sequencing of single oocytes revealed that the three genes are potentially expressed at very low amounts in such cells (Reich et al., 2012).

**Table 1:** **Summary on prion protein family expression in placenta and knockout phenotypes**.

**Gene (Protein)**	**Expression in embryonic stem cells**	**Expression in placenta**	**Placental and related phenotype in knockout mice**
*Prnp* (PrP)	Published	Published	Fetal and post-natal growth retardation. Placental abnormalities (weight, structure).
			Transcriptomic alterations. (Khalifé et al., 2011; Alfaidy et al., 2013)
*Sprn* (Shadoo)	Published	Published	Post-natal growth retardation (Daude et al., 2012).
			Transcriptomic alterations to be confirmed (knockdown experiments only, Passet et al., 2012)
*Prnd* (Doppel)	Published	Our observation	No described effect but no reported specific analysis.

*Data on gene expression analyses are derived from various sources, origin of which can be found in the article*.

Overall, these studies suggest that in most mammalian species, the prion family genes are transcribed very early in embryogenesis, in totipotent cells, and that this expression remains both in embryonic and extra-embryonic tissues at early differentiation stages (Table 1). At later developmental times, more restricted and cell-specific expression patterns have been reported, but only few studies focused on these loci. Nevertheless, these observations suggest a potential involvement of this gene family in early mammalian developmental stages and highlight its potential implication in the differentiation of the extra-embryonic tissues.

## Potential incidence of single prion protein family gene on placenta

### The *Prnp* locus

In sheep, PrP variants are strongly associated with differences in animal susceptibility to prion. The observation that some of these PrP variants confer a stronger resistance of the animal to some prion strains resulted in selection breeding strategies aiming at reducing the prevalence of the disease in the sheep population through the selection of so-called resistant *Prnp* alleles, such as ARR vs. VRQ at codons 136, 154, and 171, respectively (Clouscard et al., 1995; Jeffrey et al., 2014). While selecting ARR animals seems efficient against scrapie development, it could have negative consequences should *Prnp* or closely related genes be involved in important economical traits, since it would force the breeder not to consider selecting for interesting traits if they are associated to a sensitive (VRQ) genotype. Indeed, several articles described a potential association between the *Prnp* genotype and specific traits such as lamb birth weight (Sawalha et al., 2007b) and (post-natal) survival (Sawalha et al., 2007a; Gubbins et al., 2009). Although these are complex traits with several potential origins, a correlation between these observations and a role of PrP during placentation/early development could be suspected.

Potential implication of PrP in the placental physiology was also suggested by the observation of the deregulation of its gene expression in pathological situations. In human, one of the most studied albeit still mysterious gestational disease is preeclampsia. Preeclampsia is characterized by a *de novo* gestational hypertension and proteinuria. It is a major source of maternal and neonatal morbidity and mortality (Sibai et al., 2005). In one third of the cases, preeclampsia is accompanied with Intra-Uterine Growth Restriction (IUGR), a disease where the fetus fails to reach its genetic growth potential, often through placental dysfunction. Oxidative stress is associated with preeclampsia (Shaker and Sadik, 2013) and abnormal placenta levels of zinc and copper with preterm gestations and IUGR both in humans (Zadrozna et al., 2009; Uriu-Adams and Keen, 2010; Kambe et al., 2014) and mouse (Andrews et al., 2004; Tian et al., 2014). Placental tissues deriving from preeclamptic pregnancies were found to over-express both the *Prnp* gene mRNA and the PrP protein (Nishizawa et al., 2007; Hwang et al., 2010). However, this over-expression was not equally distributed within the tissue and appeared to be restricted to the syncytiotrophoblast (the external layer of the placental villi) and to the cytotrophoblasts (the layer of cells that underline the syncytiotrophoblast). Both in human (Bilodeau, 2014) and in a recently published preeclamptic mouse model (Doridot et al., 2013, 2014), strong links between preeclampsia and oxidative stress were described. The octapeptide region and adjacent sites, around histidines 96 and 111, of the PrP protein localized in its amino-terminal region bind several divalent metals (Jackson et al., 2001; Kramer et al., 2001; Walter et al., 2009). This ability has been associated with putative PrP functions in the binding and internalizations of ions, ferrireductase and superoxide dismutase-like activities, signal transduction and protection against oxidative stress (Brown et al., 2001; Choi et al., 2007; Bertuchi et al., 2012; Watt et al., 2012; Singh et al., 2013). Concurrently, a recent study by Alfaidy et al. depicted an involvement of PrP in the response to oxidative stress in the placenta (Alfaidy et al., 2013). It was thus hypothesized that these increased protein and mRNA PrP levels could be part of a compensatory mechanism induced by a preeclamptic status. However, beside this protective function, PrP could also play an active role in the establishment of preeclampsia as signaling pathways involving PrP have been proposed to participate or to worsen this disease. The TGFβ pathway, modulated by the prion protein (Wurm and Wechselberger, 2006), is suspected to contribute to the preeclamptic etiopathology (Stanczuk et al., 2007; Feizollahzadeh et al., 2012; Ozkan et al., 2013), possibly by altering Treg cells equilibrium (Laresgoiti-Servitje et al., 2010; Robertson et al., 2013). Alteration of the Notch-signaling pathway leads to a defect of trophoblast invasion that contributes to the pathogenesis of preeclampsia (Hunkapiller et al., 2011). Interestingly, invalidation of the PrP appears to modulate the placental Notch signaling pathway through alteration of the expression of the Jagged1 ligand (SMR et al., unpublished observation). Thus, PrP could coordinate various signal pathways in response to an oxidative stress that lead to the development of preeclampsia.

Another indirect evidence of PrP implication in placentation came from comparative transcriptomic studies of E6.5 and E7.5 FVB/N and FVB/N *Prnp*^−/−^ embryos (Khalifé et al., 2011). These data revealed that in *Prnp* gene-invalidated embryos several major metabolic pathways such as angiogenesis, cell proliferation, adhesion and movement were affected at these early developmental stages. Such pathways are important in placental physiology. Furthermore, some of the identified deregulated genes were previously described as key actors of mammalian placentation, such as Adam12 (Huppertz et al., 2006) and activin receptors (Munir et al., 2004).

A direct implication of PrP in regulating placental function and pregnancy outcomes was recently documented (Alfaidy et al., 2013). This study comparatively analyzed the reproductive performances of transgenic mice either over-expressing mouse PrP (Tga20) or knockout for *Prnp* (*Prnp*^−/−^) with those of their wild type (WT) counterpart. The litter size was decreased in Tga20 comparatively to that of *Prnp*^−/−^ or WT mice. At E17.5, the fetal and placental weights of the *Prnp*^−/−^ mice were lower than that of the two other genotypes and in their adulthood, both the *Prnp*^−/−^ and Tga20 mice had lower body weights compared to their WT counterparts. This study further highlighted a role of PrP in placental Cu homeostasis and protection against oxidative stress as well as its involvement in placental angiogenesis, two of the altered metabolic pathways identified in the above-mentioned embryonic transcriptomic study (Khalifé et al., 2011). Phenotypic analysis of *Prnp*^−/−^ mice revealed a compacted placental labyrinth structure and a disorganization of the vascular tree, disorganization also observed in Tga20 placentas. Alteration of the expression of genes specific of spongiotrophoblasts, invasive trophoblasts or related to the labyrinth branching were noticed. Trophoblastic cell mobility is an important physiological process that allows placental cells to invade uterine tissue, contributing to the anchorage of the placenta, to the regulation of decidual angiogenesis and to the remodeling of the maternal spiral arteries. This mechanism is complex and involves different cell types and signaling pathways (Gasperowicz and Otto, 2008; Knofler, 2010, for recent reviews). PrP might also be directly involved in angiogenesis (Turu et al., 2008) and hematopoiesis (Palmqvist et al., 2007). This might be related to its localization in caveolae and interaction with Caveolin-1, known to be involved in angiogenesis (Griffoni et al., 2000; Massimino et al., 2002).

Overall these studies highlighted a role of PrP during placentation. Deregulation of the expression of the gene and differences in PrP genotypes were associated with placental perturbations (Table 1). However, these events did not result in overt phenotypes, suggesting either that PrP function in this tissue is not crucial enough for its development to induce embryonic distress or that compensatory mechanisms exist.

### The *Prnd* and *Sprn* loci

The *Prnd* gene has been invalidated in two different mouse genetic backgrounds, 129/Ola (Paisley et al., 2004) and a mixed C57BL6/CBA one (Behrens et al., 2002). In both experiments, knockout males suffered from severe subfertility involving a defect in acrosome biogenesis. These males also suffered from other sperm abnormalities that varied between the two reports probably in relation with the different genetic background. Because of this male phenotype, a knockout transgenic line could not be propagated by classical breeding and homozygous knockout animals were only routinely obtained using heterozygous males. In both articles, reproduction of the knockout females were reported to be normal in terms of fertility and litter sizes. Although not specifically studied, none of these reports described other associated phenotypes, suggesting either that Doppel has no role during early embryogenesis or that, should it be involved in placentation, its gene invalidation does not induce drastic alterations (Table 1). However, as Doppel is expressed in mouse extra-embryonic tissues (our unpublished observation), more dedicated analyses should probably be conducted before formally excluding it from the list of proteins involved in the physiology of the placenta. The α-helical region of the Doppel protein binds copper (Qin et al., 2003), but this interaction does not appear to induce the protein internalization, as it is observed for PrP (Cereghetti et al., 2004). It was also reported that Doppel expression might exacerbate oxidative damage (Wong et al., 2001), but this property is discussed (Qin et al., 2003). Thus, analysis of a putative role of Doppel in oxidative stress homeostasis during placental development would be worth looking at.

The *Sprn* gene invalidation was also recently obtained by homologous recombination in 129Pas ES cells (Daude et al., 2012). These cells were used to derive C57BL/129 and FVB/NCr/129 *Sprn*^−/−^ mice that were reported to have no gross morphological alterations and to be fertile (Daude et al., 2012). However, 8–16 days old knockout females from the first genetic background were found to have slightly lighter body weights compared to their wild-type counterparts, while knockout males of both genetic backgrounds had a similar weight phenotype at older ages, from day 28 till 42 for C57BL/129 and from day 22 to 50 for FVB/NCr/129 *Sprn*^−/−^ mice, respectively. Overall this subtle alteration resulted in a weight decrement of about 9%. In a following up review, the authors suggested that the origin of this phenotype could be related to the natural expression of Shadoo in hypothalamic neurons, a structure associated with the control of feeding behavior (Daude and Westaway, 2012). Several other explanations could also be proposed. A mammary defect resulting in modified lactation ability of the knockout females would also induce growth retardations; however such a phenotype would probably affect all pups similarly. Interestingly, uteroplacental insufficiency and altered maternal nutrition during gestation have also been associated with sex-specific post-natal development defects of rat offsprings (Howie et al., 2012; Wadley et al., 2013). It could thus be also hypothesized that the body weight phenotype described for the *Sprn*^−/−^ pups arises from a defect in the placentation process. In line with this suggestion is the observation that the mean litter size of these *Sprn*^−/−^ females is smaller than that of their *Sprn*^+/−^ counterparts (Table S1 in Daude et al., 2012). Transcriptomic analyses performed on FVB/N *Sprn*-knockdown embryos at E6.5 and E7.5 also revealed the differential expression of around 60 transcripts associated with cellular movement and development and with differentiation of the hematological system. Interestingly, some of them, such as the prolactin-related genes, pointed to a transcriptomic deregulation affecting precisely the trophectoderm-derived compartment (Passet et al., 2012, 2013). Regulation of cellular metal uptake by PrP is probably regulated through the interaction of this protein with other membrane-bound partners such as the lipoprotein-receptor-related protein LRP1 (Taylor and Hooper, 2007) or the glutamate receptor AMPA (Watt et al., 2012) and, as already discussed, this function was shown to be important for placentation (Alfaidy et al., 2013). Although not known to bind divalent metals, Shadoo might also indirectly regulate oxidative stress homeostasis regulatory processes through interaction with PrP and/or other proteins from their shared interactome (Watts et al., 2009). This hypothesis has yet to be substantiated by experimental evidences. Overall, these observations are compatible with a gene dosage effect of *Sprn* expression on the ectoplacental cone differentiation and of a potential incidence of the genetic background on the resulting phenotype, as already mentioned for *Prnd* (Table 1).

## Co-invalidation of several members of the prion protein gene family

As already mentioned, the absence of a strong phenotype in mammals invalidated for a single member of the prion protein gene family has led to the hypothesis of the existence of a biological functional redundancy with another host-encoded protein. This model was first developed for the implication of PrP in the central nervous system (Shmerling et al., 1998). Such proteins should share with PrP an overlapping pattern of expression, at least at some developmental time points, and related biological properties. The two PrP related proteins, Shadoo and Doppel, were suspected to play this role.

### *Prnd* and *Prnp* co-invalidation

Doppel is unlikely to compensate for the lack of PrP based on its adult pattern of expression, mainly restricted to non-nervous tissues and the male gonads, and on its biological properties in terms of neurotoxicity. Indeed, induced ectopic activation of the *Prnd* gene expression in the adult nervous system of some *Prnp*-knockout mice was shown to be neurotoxic (Moore et al., 1999, 2001; Rossi et al., 2001; Anderson et al., 2004) and antagonized by full length PrP expression (Yamaguchi et al., 2004). However, because Doppel and PrP are both expressed during embryogenesis, it remained possible that these two proteins share crucial properties at earlier developmental stages. To investigate a potential compensation by Doppel for the absence of PrP, both loci were co-invalidated in ES cells, taking advantage of their physical close linkage (Paisley et al., 2004). The resulting animals were reported to develop normally into adulthood with no overt phenotype beside an infertility syndrome in the double-knockout males indistinguishable from that observed in their single *Prnd*-knockout counterparts. Thus, this experiment strongly suggests that Doppel and PrP share no obvious compensatory mechanisms. The reproductive performances of these mice, apart that of the males, were however not specifically assessed.

### *Sprn* and *Prnp* co-invalidation

The co-invalidation of *Sprn* and *Prnp* was achieved using transgenic lines derived from targeted 129Sv/Pas ES cells. Double knockout animals were obtained by crossing *Sprn*^−/−^ with FVB.129-*Prnp*^−/−^ mice (Daude et al., 2012). The resulting mice were viable, fertile and produced at expected Mendelian ratios. No overt phenotype was reported, even in aged individuals, and the above-discussed lighter body-weight of the single *Sprn*^−/−^ mice not mentioned in these animals (Daude et al., 2012). Altogether, this work strongly argued against compensation or redundancy between Shadoo and PrP in the adult nervous system and during embryogenesis (Daude and Westaway, 2012).

However, this conclusion drastically contrasts with previous experiments aimed at lowering the expression of the *Sprn* gene in various genetic backgrounds by RNA interference, RNAi (Young et al., 2009; Passet et al., 2012). Indeed, using lentiviral ShRNA-delivery in mouse embryos, *Sprn* gene knockdown resulted in early embryonic lethality, between E8 and E11, in an FVB/N.129-*Prnp*^−/−^ genetic background that was not observed in FVB/N mice (Young et al., 2009). This lethality was associated with a developmental defect of the ectoplacental cone and with important hemorrhage surrounding the embryos (Passet et al., 2012). The restriction of the lentiviral infection to the trophoblastic cell lineage resulted in a similar lethal phenotype (Passet et al., 2012). Consistently, embryos invalidated for *Prnp* and/or knockdown for *Sprn* (Khalifé et al., 2011; Passet et al., 2013) display alterations of the expression of genes involved in placental angiogenesis, such as angiopoietin genes (Geva et al., 2005), cathepsins (Screen et al., 2008) and matrix metalloproteinases (Fontana et al., 2012). It would be of interest to assess in which extent, inactivation of Shadoo and co-inactivation with PrP affects Notch signaling in regards of PrP invalidation alone and consistently with the crucial role of this pathway for mouse placental fetal angiogenesis and proper trophoblast cell type specification (Gasperowicz and Otto, 2008). PrP expression is positively regulated by MEK and p38 MAPK kinases (Wang et al., 2005) and it is noteworthy that Mek-1 deficient mice die at early embryonic stages, around E10.5, from an angiogenesis defect of the placenta (Giroux et al., 1999). This age is reminiscent of that of the lethality occurring in *Prnp*^−/−^*Sprn*-knockdown embryos (Young et al., 2009) and it would be of interest to also assess the effect of Mek-1 on the transcriptional regulation of *Sprn* in a regular context and in the absence of PrP.

It remains that this RNAi strategy yielded seemingly contradictory result compared to the classical knockout approach. Several explanations were proposed to conciliate these observations (Daude and Westaway, 2012; Passet et al., 2013). It was suggested that the RNAi approach may induce a potential artifact resulting from either an off target effect of the two used shRNA only phenotypically visible in *Prnp*-knockout embryos or a toxicity associated with an shRNA over expression in single *Prnp* or double *Prnp*/*Sprn* knockout genotypes, linked to the role of PrP and potentially Shadoo in the regulation of the RISC complex (Gibbings et al., 2012). Alternative hypotheses may also be proposed, such as a higher susceptibility of the double knockout genotype to external stresses, such as lentiviral infection. Furthermore, even if the knockdown of a gene can result in phenotypic outcomes comparable to that of the knockout (De Souza et al., 2006), haplo-insufficiency may on the opposite induce stronger phenotypes than the complete gene loss, as described for Dicer1, a key regulator of microRNA biogenesis (Lambertz et al., 2010).

The use of different genetic backgrounds is also known to greatly influence the phenotype of genetically engineered mouse (Doetschman, 2009). Although cautions were taken in that respect in the knockout experiments (see comments in Daude and Westaway, 2012), this strategy involves inbreeding crosses over several generations. Doing so, starting from different genetic backgrounds [129Pas for the ES cells, C57BL6 for the first established knockout line that was then backcrossed in FVB/NCr (Daude et al., 2012)], a selection process might take place that progressively eliminates unfavorable allelic combinations. This could also lead to potential miss-association between (absence of) a phenotype and the targeted gene, as recently exemplified in the case of PrP where abnormalities suspected to result from this gene invalidation actually originated from co-segregating 129Sv-derived loci (Nuvolone et al., 2013; Striebel et al., 2013). Such a mechanism will not occur using an RNAi approach, potentially leading to stronger associated phenotypes. One way to avoid these potential artifacts would be to use the new custom-designed DNA scissors to invalidate these loci in pure genetic backgrounds (Gaj et al., 2013).

On this basis, we have engineered *Sprn* knockout mice through the use of the Zinc Finger Nuclease (ZFN) technology. Our preliminary results indicate that, as reported by Daude et al. (2012), FVB/N *Sprn*^−/−^ and FVB/N.129 *Prnp*^−/−^/*Sprn*^−/−^ mice are viable. However, these transgenic mice suffer from various defects that are under scrutiny. In both cases, pups exhibit a growth retardation that can be attributed, for part of it, to a mammary defect of the knockout females, probably associated with *Sprn* invalidation. The FVB/N.129 *Prnp*^−/−^/*Sprn*^−/−^ mice are also characterized by increased embryonic and perinatal lethality rates compared to FVB/N or FVB/N.129 *Prnp*^−/−^ controls, the extent of which is under analysis. Finally, some phenotypic differences in the survival of the mice at the past weaning stage between FVB/N *Sprn*^−/−^ and FVB/N.129 *Prnp*^−/−^/*Sprn*^−/−^ mice appear to result from the 129 genetic contribution in the double knockout mice. Altogether, these preliminary and unpublished observations are consistent with a role of these two related proteins in placentation. They further reassert the importance of the influence of the genetic background and suggest the contribution of other yet unknown loci in the phenotypic consequences of the *Prnp* and *Sprn* invalidations.

A remaining issue concerns the discrepancy observed between the knockout and the knockdown experiments. We could exclude a specific susceptibility of the FVB/N.129 *Prnp*^−/−^/*Sprn*^−/−^ mice toward lentiviral infection *per se*. Three other hypothesis are currently tested: (i) an impact of ShRNA over-expression in FVB/N.129 *Prnp*^−/−^/*Sprn*^−/−^ embryos in relation with a potential function of these proteins in miRNA regulation, (ii) an off-target effect of the anti-*Sprn* ShRNAs used in Young et al. (2009) that would down-regulate the expression of a protein leading to a detrimental effect on the development of the ecoplacental cone in FVB/N.129 *Prnp*^−/−^ and/or FVB/N.129 *Prnp*^−/−^/*Sprn*^−/−^ mice or (iii) a genetic adaptation of the double-knockout embryos to the lack of these two proteins highlighting other loci able to compensate for the lack of them. Pursuing this analysis will thus undoubtedly increase our knowledge on early mammalian embryogenesis.

## Similarities of the signaling pathways associated with the prion protein family in placentation and in Zebrafish early embryogenesis

As mentioned before, the biological roles of the PrP, of the related Shadoo and to a lesser extend of the Doppel proteins still remain elusive in mammals. Investigations on the PrP biological role were also performed on more distantly species such as Zebrafish. Again, the outcome of these studies surprisingly differed according to the experimental approaches used, morpholinos or knockout, somehow recalling the situation described in mouse. Morpholino-induced downregulation of PrP1 or PrP2 resulted in high mortality of the depleted early embryos. Lethality occurred at developmental stages related to the respective two genes' spatio-temporal patterns of expression, and was associated with deficient morphogenic cell movements and induced apoptotic cell death (Malaga-Trillo et al., 2009; Nourizadeh-Lillabadi et al., 2010). Surviving PrP2- (Nourizadeh-Lillabadi et al., 2010) or PrP1- (Kaiser et al., 2012) depleted embryos also highlighted a neuroprotective role of these proteins. In contrast to PrP-2 morpholino experiments, the knockout of this gene by a ZFN-based approach resulted in no overt developmental phenotypes (Fleisch et al., 2013). This apparent discrepancy was attributed in part to an off target effect of the used morpholinos. In contrast, PrP1-knockdown by morpholinos can be considered as specific as the phenotype can be partially rescued by the injection of PrP1 or PrP2 mRNA.

Interestingly, the function of PrP1 in early embryonic development of the Zebrafish could be partly rescued in depleted embryos by the injection of mammalian PrP-encoding mRNAs, suggesting evolutionary conservation of some of the protein functions (Chiesa and Harris, 2009; Malaga-Trillo and Sempou, 2009; Malaga-Trillo et al., 2009; Solis et al., 2013). Besides, in mouse *Prnp*^−/−^ embryos, transcriptomic analyses revealed similarities with PrP1-depleted Zebrafish eggs by highlighting the over-expression of genes encoding proteins with proteolysis activities that could modulate cellular adhesion and proliferation as well as extracellular matrix deposition (Khalifé et al., 2011). The same pathways were also deregulated in Shadoo-depleted embryos, suggesting that this protein shares some of these PrP-functions at least in mammals (Passet et al., 2012). In Zebrafish, the PrP1-depleted phenotype is associated with an abnormal intracellular processing and/or transport of E-cadherin, potentially resulting from a modulation of the Fyn signaling pathway (Malaga-Trillo et al., 2009). Similarly in mammals, PrP has been linked to the activation of Src kinases, including Fyn (Mouillet-Richard et al., 2000), while in early mouse embryos, both *Prnp* and *Sprn* downregulations induced protocadherin and cadherin transcriptomic alterations (Khalifé et al., 2011; Passet et al., 2012). In mouse embryos, the trophectoderm is the first differentiated tissue to form, with cells requiring complex adhesive structures and invasive capacities. Migrating trophoblast cells are first observed at E6.5 (Croy et al., 2012). Thus, the alterations of cell adhesion pathways in *Sprn* and *Prnp* invalidated mice, similar to the alterations exhibited by PrP1-depleted zebrafish, are very likely to affect this specialized tissue at these early embryonic stages.

## Conclusion—ongoing or potential future directions

Current data clearly indicate that the PrP protein has an important role in early mammalian embryogenesis through its implication in placental physiology. The mechanistic behind these observations has yet to be clearly deciphered and could involve several, not mutually exclusive, processes such as an involvement in cell adhesion and angiogenesis as well as a role in the control and response to oxidative stress. A similar implication of Shadoo has also been suggested by several studies but whether this protein acts through identical, complementary or unrelated biological pathways with those of PrP remains a debatable question. The properties of Doppel and its pattern of expression make it an interesting potential partner, but no evidence so far has pointed to this protein as being a key actor of placental physiology. Taking into account the phenotypes observed through the modulation of the expression of these genes in placenta and the affected pathways, the knowledge accumulated on the function of these proteins in this tissue could be of particular interest in regards with their more and more recognized implication in cancers. A crosstalk between these so far independent studies would be of great interest with potential benefits for human and animal health.

A yet poorly investigated question is the relation between these proteins and the biology of mitochondria. PrP depletion was shown to result in reduced mitochondrial numbers and unusual mitochondrial morphology with increased diameters and poorly defined and sparse cristae in several mouse tissues (Miele et al., 2002). PrP depletion induced elevated mitochondrial manganese superoxide dismutase activity, manganese-induced mitochondrial depolarization and reactive oxygen species generation. Brains of Prion-infected mice have reduced cytochrome C oxidase and manganese superoxide dismutase activities and contain swelling mitochondria. Oxidative stress conditions can induce PrP localization in raft-like microdomains of the mitochondria membranes, resulting in loss of this membrane potential. Impaired mitochondrial function is associated with perturbations of mouse placental (Wakefield et al., 2011) and potentially with preeclampsia (Shi et al., 2013; Doridot et al., 2014) developments. Thus, it is tempting to suggest that oxidative stress conditions and preeclampsia through its induced over-expression of PrP, might trigger the delocalization of some PrP proteins in sub-domains of the mitochondria membrane resulting in induced mitochondria dysfunctions and placentation defects. Similar implication of the other prion protein family members is a yet-to-be explored direction for future experimentations.

One difficulty to delineate the role of these proteins might be related with their mode of action. As nicely highlighted by Alfaidy et al. (2013), these proteins may act as gatekeeper of cellular integrity and thus, in the absence of specific insults such as oxidative stress conditions, their absence might not yield strong phenotypes. Challenging through diverse approaches the existing panel of created animal models with altered expression of these genes would reveal such roles.

It was also suggested that these proteins, which have diverse and large interactomes, may participate to versatile signaling scaffold complexes, allowing activation of various biological pathways. As such, one might expect this function to be revealed by invalidation of their encoding genes unless (i) biological redundancy exists, (ii) the involved biological pathways are associated with non-essential functions or related to response to various stress as discussed just before, (iii) their absence may be, at least partially, compensated through selection of specific genetic environment. This latter hypothesis is attractive as it would explain the different outcomes of the knockout of these genes according to the genetic background, as observed for Doppel, and/or to the methodology used, as observed for Shadoo. It would also reconcile the discrepancy between *in vitro* data, suggesting that these proteins have key biological functions in crucial pathways such as stem cell homeostasis, with *in vivo* observations, which appear to deny or limit their importance. The classical knockout process involves the establishment of transgenic lines in mixed genetic backgrounds, allowing the selection process of the less detrimental genetic combinations. New approaches now exist to assess gene invalidation in controlled genetic backgrounds, such as the use of endonucleases (Wijshake et al., 2014). The implementation of this approach to target the prion gene family in different mouse strains might help us to better understand the role of each of these proteins and their potential biological redundancy. Such experiments are currently ongoing and their conclusions much awaited.

## Author contributions

All authors contributed to the conception of this review, revised the manuscript and approved the submitted version.

### Conflict of interest statement

The authors declare that the research was conducted in the absence of any commercial or financial relationships that could be construed as a potential conflict of interest.

## References

[B1] Alfaidy N. Chauvet S. Donadio-Andrei S. Salomon A. Saoudi Y. Richaud P.. (2013). Prion protein expression and functional importance in developmental angiogenesis: role in oxidative stress and copper homeostasis. Antioxid. Redox Signal. 18, 400–411. 10.1089/ars.2012.4637 22861352

[B2] Anderson L. Rossi D. Linehan J. Brandner S. Weissmann C. (2004). Transgene-driven expression of the Doppel protein in Purkinje cells causes Purkinje cell degeneration and motor impairment. Proc. Natl. Acad. Sci. U.S.A. 101, 3644–3649. 10.1073/pnas.0308681101 15007176 PMC373516

[B3] Andrews G. K. Wang H. Dey S. K. Palmiter R. D. (2004). Mouse zinc transporter 1 gene provides an essential function during early embryonic development. Genesis 40, 74–81. 10.1002/gene.20067 15452870

[B4] Behrens A. Genoud N. Naumann H. Rulicke T. Janett F. Heppner F. L.. (2002). Absence of the prion protein homologue Doppel causes male sterility. EMBO J. 21, 3652–3658. 10.1093/emboj/cdf386 12110578 PMC125402

[B5] Bertuchi F. R. Bourgeon D. M. Landemberger M. C. Martins V. R. Cerchiaro G. (2012). PrPC displays an essential protective role from oxidative stress in an astrocyte cell line derived from PrPC knockout mice. Biochem. Biophys. Res. Commun. 418, 27–32. 10.1016/j.bbrc.2011.12.098 22222374

[B6] Bilodeau J. F. (2014). Review: maternal and placental antioxidant response to preeclampsia —impact on vasoactive eicosanoids. Placenta 35(Suppl.), S32–S38. 10.1016/j.placenta.2013.11.013 24333047

[B7] Brown D. R. Clive C. Haswell S. J. (2001). Antioxidant activity related to copper binding of native prion protein. J. Neurochem. 76, 69–76. 10.1046/j.1471-4159.2001.00009.x 11145979

[B8] Büeler H. Aguzzi A. Sailer A. Greiner R. A. Autenried P. Aguet M.. (1993). Mice devoid of PrP are resistant to scrapie. Cell 73, 1339–1347. 10.1016/0092-8674(93)90360-3 8100741

[B9] Büeler H. Fischer M. Lang Y. Bluethmann H. Lipp H. P. Dearmond S. J.. (1992). Normal development and behaviour of mice lacking the neuronal cell-surface PrP protein. Nature 356, 577–582. 10.1038/356577a0 1373228

[B10] Cereghetti G. M. Negro A. Vinck E. Massimino M. L. Sorgato M. C. Van Doorslaer S. (2004). Copper(II) binding to the human Doppel protein may mark its functional diversity from the prion protein. J. Biol. Chem. 279, 36497–36503. 10.1074/jbc.M404341200 15218028

[B11] Chadi S. Young R. Le Guillou S. Tilly G. Bitton F. Martin-Magniette M. L.. (2010). Brain transcriptional stability upon prion protein-encoding gene invalidation in zygotic or adult mouse. BMC Genomics 11:448. 10.1186/1471-2164-11-448 20649983 PMC3091645

[B12] Chiesa R. Harris D. A. (2009). Fishing for prion protein function. PLoS Biol. 7:e75. 10.1371/journal.pbio.1000075 19338390 PMC2661967

[B13] Choi C. J. Anantharam V. Saetveit N. J. Houk R. S. Kanthasamy A. Kanthasamy A. G. (2007). Normal cellular prion protein protects against manganese-induced oxidative stress and apoptotic cell death. Toxicol. Sci. 98, 495–509. 10.1093/toxsci/kfm099 17483122 PMC3407037

[B14] Clouscard C. Beaudry P. Elsen J. M. Milan D. Dussaucy M. Bounneau C.. (1995). Different allelic effects of the codons 136 and 171 of the prion protein gene in sheep with natural scrapie. J. Gen. Virol. 76(Pt 8), 2097–20101. 10.1099/0022-1317-76-8-2097 7636494

[B15] Croy B. A. Chen Z. Hofmann A. P. Lord E. M. Sedlacek A. L. Gerber S. A. (2012). Imaging of vascular development in early mouse decidua and its association with leukocytes and trophoblasts. Biol. Reprod. 87, 125. 10.1095/biolreprod.112.102830 22954796 PMC3509781

[B16] Daude N. Westaway D. (2012). Shadoo/PrP (*Sprn*(0/0) /*Prnp*(0/0)) double knockout mice: more than zeroes. Prion 6, 420–424. 10.4161/pri.21867 22929230 PMC3510864

[B17] Daude N. Wohlgemuth S. Brown R. Pitstick R. Gapeshina H. Yang J.. (2012). Knockout of the prion protein (PrP)-like *Sprn* gene does not produce embryonic lethality in combination with PrP(C)-deficiency. Proc. Natl. Acad. Sci. U.S.A. 109, 9035–9040. 10.1073/pnas.1202130109 22619325 PMC3384183

[B18] De Souza A. T. Dai X. Spencer A. G. Reppen T. Menzie A. Roesch P. L.. (2006). Transcriptional and phenotypic comparisons of Ppara knockout and siRNA knockdown mice. Nucleic Acids Res. 34, 4486–4494. 10.1093/nar/gkl609 16945951 PMC1636368

[B19] Doetschman T. (2009). Influence of genetic background on genetically engineered mouse phenotypes. Methods Mol. Biol. 530, 423–433. 10.1007/978-1-59745-471-1_23 19266333 PMC2805848

[B20] Donadio S. Alfaidy N. De Keukeleire B. Micoud J. Feige J. J. Challis J. R.. (2007). Expression and localization of cellular prion and COMMD1 proteins in human placenta throughout pregnancy. Placenta 28, 907–911. 10.1016/j.placenta.2006.11.006 17254632

[B21] Doridot L. D. Chatre L. D. Ducat A. Vilotte J. L. Lombes A. Mehats C.. (2014). Nitroso-redox balance and mitochondrial homeostasis are regulated by Stox1, a pre-eclampsia associated gene. Antioxid. Redox Signal. 21, 819–834. 10.1089/ars.2013.5661 24738702 PMC4116089

[B22] Doridot L. Passet B. Méhats C. Rigourd V. Barbaux S. Ducat A.. (2013). Preeclampsia-like symptoms induced in mice by fetoplacental expression of STOX1 are reversed by aspirin treatment. Hypertension 61, 662–668. 10.1161/HYPERTENSIONAHA.111.202994 23357179

[B23] Ehsani S. Tao R. Pocanschi C. L. Ren H. Harrison P. M. Schmitt-Ulms G. (2011). Evidence for retrogene origins of the prion gene family. PLoS ONE 6:e26800. 10.1371/journal.pone.0026800 22046361 PMC3203146

[B24] Feizollahzadeh S. Taheripanah R. Khani M. Farokhi B. Amani D. (2012). Promoter region polymorphisms in the transforming growth factor beta-1 (TGFbeta1) gene and serum TGFbeta1 concentration in preeclamptic and control Iranian women. J. Reprod. Immunol. 94, 216–221. 10.1016/j.jri.2012.02.006 22503347

[B25] Fleisch V. C. Leighton P. L. Wang H. Pillay L. M. Ritzel R. G. Bhinder G.. (2013). Targeted mutation of the gene encoding prion protein in zebrafish reveals a conserved role in neuron excitability. Neurobiol. Dis. 55, 11–25. 10.1016/j.nbd.2013.03.007 23523635

[B26] Fontana V. Coll T. A. Sobarzo C. M. Tito L. P. Calvo J. C. Cebral E. (2012). Matrix metalloproteinase expression and activity in trophoblast-decidual tissues at organogenesis in CF-1 mouse. J. Mol. Histol. 43, 487–496. 10.1007/s10735-012-9429-8 22714107

[B27] Gaj T. Gersbach C. A. Barbas C. F. 3rd. (2013). ZFN, TALEN, and CRISPR/Cas-based methods for genome engineering. Trends Biotechnol. 31, 397–405. 10.1016/j.tibtech.2013.04.004 23664777 PMC3694601

[B28] Gasperowicz M. Otto F. (2008). The notch signalling pathway in the development of the mouse placenta. Placenta 29, 651–659. 10.1016/j.placenta.2008.06.004 18603295

[B29] Geva E. Ginzinger D. G. Moore D. H. 2nd. Ursell P. C. Jaffe R. B. (2005). *In utero* angiopoietin-2 gene delivery remodels placental blood vessel phenotype: a murine model for studying placental angiogenesis. Mol. Hum. Reprod. 11, 253–260. 10.1093/molehr/gah159 15734895

[B30] Gibbings D. Leblanc P. Jay F. Pontier D. Michel F. Schwab Y.. (2012). Human prion protein binds Argonaute and promotes accumulation of microRNA effector complexes. Nat. Struct. Mol. Biol. 19, 517–524. 10.1038/nsmb.2273 22484317

[B31] Giroux S. Tremblay M. Bernard D. Cardin-Girard J. F. Aubry S. Larouche L.. (1999). Embryonic death of Mek1-deficient mice reveals a role for this kinase in angiogenesis in the labyrinthine region of the placenta. Curr. Biol. 9, 369–372. 10.1016/S0960-9822(99)80164-X 10209122

[B32] Griffoni C. Spisni E. Santi S. Riccio M. Guarnieri T. Tomasi V. (2000). Knockdown of caveolin-1 by antisense oligonucleotides impairs angiogenesis *in vitro* and *in vivo*. Biochem. Biophys. Res. Commun. 276, 756–761. 10.1006/bbrc.2000.3484 11027543

[B33] Gubbins S. Cook C. J. Hyder K. Boulton K. Davis C. Thomas E.. (2009). Associations between lamb survival and prion protein genotype: analysis of data for ten sheep breeds in Great Britain. BMC Vet. Res. 5:3. 10.1186/1746-6148-5-3 19159456 PMC2637852

[B34] Haigh C. L. Marom S. Y. Collins S. J. (2010). Copper, endoproteolytic processing of the prion protein and cell signalling. Front. Biosci. (Landmark Ed). 15, 1086–1104. 10.2741/3663 20515743

[B35] Howie G. J. Sloboda D. M. Vickers M. H. (2012). Maternal undernutrition during critical windows of development results in differential and sex-specific effects on postnatal adiposity and related metabolic profiles in adult rat offspring. Br. J. Nutr. 108, 298–307. 10.1017/S000711451100554X 22018052

[B36] Hunkapiller N. M. Gasperowicz M. Kapidzic M. Plaks V. Maltepe E. Kitajewski J.. (2011). A role for Notch signaling in trophoblast endovascular invasion and in the pathogenesis of pre-eclampsia. Development 138, 2987–2998. 10.1242/dev.066589 21693515 PMC3119307

[B37] Huppertz B. Bartz C. Kokozidou M. (2006). Trophoblast fusion: fusogenic proteins, syncytins and ADAMs, and other prerequisites for syncytial fusion. Micron 37, 509–517. 10.1016/j.micron.2005.12.011 16497505

[B38] Hwang H. S. Park S. H. Park Y. W. Kwon H. S. Sohn I. S. (2010). Expression of cellular prion protein in the placentas of women with normal and preeclamptic pregnancies. Acta Obstet. Gynecol. Scand. 89, 1155–1161. 10.3109/00016349.2010.498497 20804341

[B39] Jackson G. S. Murray I. Hosszu L. L. Gibbs N. Waltho J. P. Clarke A. R.. (2001). Location and properties of metal-binding sites on the human prion protein. Proc. Natl. Acad. Sci. U.S.A. 98, 8531–8535. 10.1073/pnas.151038498 11438695 PMC37470

[B40] Jeffrey M. Martin S. Chianini F. Eaton S. Dagleish M. P. González L. (2014). Incidence of infection in *Prnp* ARR/ARR sheep following experimental inoculation with or natural exposure to classical scrapie. PLoS ONE 9:e91026. 10.1371/journal.pone.0091026 24614120 PMC3948952

[B41] Kaiser D. M. Acharya M. Leighton P. L. Wang H. Daude N. Wohlgemuth S.. (2012). Amyloid beta precursor protein and prion protein have a conserved interaction affecting cell adhesion and CNS development. PLoS ONE 7:e51305. 10.1371/journal.pone.0051305 23236467 PMC3517466

[B42] Kambe T. Hashimoto A. Fujimoto S. (2014). Current understanding of ZIP and ZnT zinc transporters in human health and diseases. Cell. Mol. Life Sci. [Epub ahead of print]. 10.1007/s00018-014-1617-0 24710731 PMC11113243

[B43] Khalifé M. Young R. Passet B. Halliez S. Vilotte M. Jaffrezic F.. (2011). Transcriptomic analysis brings new insight into the biological role of the prion protein during mouse embryogenesis. PLoS ONE 6:e23253. 10.1371/journal.pone.0023253 21858045 PMC3156130

[B44] Knofler M. (2010). Critical growth factors and signalling pathways controlling human trophoblast invasion. Int. J. Dev. Biol. 54, 269–280. 10.1387/ijdb.082769mk 19876833 PMC2974212

[B45] Kramer M. L. Kratzin H. D. Schmidt B. Romer A. Windl O. Liemann S.. (2001). Prion protein binds copper within the physiological concentration range. J. Biol. Chem. 276, 16711–16719. 10.1074/jbc.M006554200 11278306

[B46] Krejciova Z. Pells S. Cancellotti E. Freile P. Bishop M. Samuel K.. (2011). Human embryonic stem cells rapidly take up and then clear exogenous human and animal prions *in vitro*. J. Pathol. 223, 635–645. 10.1002/path.2832 21341268

[B47] Kubosaki A. Ueno A. Matsumoto Y. Doi K. Saeki K. Onodera T. (2000). Analysis of prion protein mRNA by *in situ* hybridization in brain and placenta of sheep. Biochem. Biophys. Res. Commun. 273, 890–893. 10.1006/bbrc.2000.3035 10891342

[B48] Lambertz I. Nittner D. Mestdagh P. Denecker G. Vandesompele J. Dyer M. A.. (2010). Monoallelic but not biallelic loss of Dicer1 promotes tumorigenesis *in vivo*. Cell Death Differ. 17, 633–641. 10.1038/cdd.2009.202 20019750 PMC2892162

[B49] Laresgoiti-Servitje E. Gómez-López N. Olson D. M. (2010). An immunological insight into the origins of pre-eclampsia. Hum. Reprod. Update 16, 510–524. 10.1093/humupd/dmq007 20388637

[B50] Lee Y. J. Baskakov I. V. (2013). The cellular form of the prion protein is involved in controlling cell cycle dynamics, self-renewal, and the fate of human embryonic stem cell differentiation. J. Neurochem. 124, 310–322. 10.1111/j.1471-4159.2012.07913.x 22860629 PMC3505810

[B51] Linden R. Martins V. R. Prado M. A. Cammarota M. Izquierdo I. Brentani R. R. (2008). Physiology of the prion protein. Physiol. Rev. 88, 673–728. 10.1152/physrev.00007.2007 18391177

[B52] Lopes M. H. Santos T. G. (2012). Prion potency in stem cells biology. Prion 6, 142–146. 10.4161/pri.19035 22437733 PMC7082090

[B53] Malaga-Trillo E. Sempou E. (2009). PrPs: proteins with a purpose: lessons from the zebrafish. Prion 3, 129–133. 10.4161/pri.3.3.9651 19786844 PMC2802776

[B54] Malaga-Trillo E. Solis G. P. Schrock Y. Geiss C. Luncz L. Thomanetz V.. (2009). Regulation of embryonic cell adhesion by the prion protein. PLoS Biol. 7:e55. 10.1371/journal.pbio.1000055 19278297 PMC2653553

[B55] Mallucci G. R. Ratte S. Asante E. A. Linehan J. Gowland I. Jefferys J. G.. (2002). Post-natal knockout of prion protein alters hippocampal CA1 properties, but does not result in neurodegeneration. EMBO J. 21, 202–210. 10.1093/emboj/21.3.202 11823413 PMC125833

[B56] Manson J. C. Clarke A. R. McBride P. A. Mcconnell I. Hope J. (1994). PrP gene dosage determines the timing but not the final intensity or distribution of lesions in scrapie pathology. Neurodegeneration 3, 331–340. 7842304

[B57] Manson J. West J. D. Thomson V. McBride P. Kaufman M. H. Hope J. (1992). The prion protein gene: a role in mouse embryogenesis? Development 115, 117–122. 1353438 10.1242/dev.115.1.117

[B58] Massimino M. L. Griffoni C. Spisni E. Toni M. Tomasi V. (2002). Involvement of caveolae and caveolae-like domains in signalling, cell survival and angiogenesis. Cell. Signal. 14, 93–98. 10.1016/S0898-6568(01)00232-7 11781132

[B59] Miele G. Alejo Blanco A. R. Baybutt H. Horvat S. Manson J. Clinton M. (2003). Embryonic activation and developmental expression of the murine prion protein gene. Gene Expr. 11, 1–12. 10.3727/000000003783992324 12691521 PMC5991155

[B60] Miele G. Jeffrey M. Turnbull D. Manson J. Clinton M. (2002). Ablation of cellular prion protein expression affects mitochondrial numbers and morphology. Biochem. Biophys. Res. Commun. 291, 372–377. 10.1006/bbrc.2002.6460 11846415

[B61] Miranda A. Pericuesta E. Ramirez M. A. Gutierrez-Adan A. (2011). Prion protein expression regulates embryonic stem cell pluripotency and differentiation. PLoS ONE 6:e18422. 10.1371/journal.pone.0018422 21483752 PMC3070729

[B62] Moore R. C. Lee I. Y. Silverman G. L. Harrison P. M. Strome R. Heinrich C.. (1999). Ataxia in prion protein (PrP)-deficient mice is associated with upregulation of the novel PrP-like protein doppel. J. Mol. Biol. 292, 797–817. 10.1006/jmbi.1999.3108 10525406

[B63] Moore R. C. Xiang F. Monaghan J. Han D. Zhang Z. Edstrom L.. (2001). Huntington disease phenocopy is a familial prion disease. Am. J. Hum. Genet. 69, 1385–1388. 10.1086/324414 11593450 PMC1235549

[B64] Mouillet-Richard S. Ermonval M. Chebassier C. Laplanche J. L. Lehmann S. Launay J. M.. (2000). Signal transduction through prion protein. Science 289, 1925–1928. 10.1126/science.289.5486.1925 10988071

[B65] Munir S. Xu G. Wu Y. Yang B. Lala P. K. Peng C. (2004). Nodal and ALK7 inhibit proliferation and induce apoptosis in human trophoblast cells. J. Biol. Chem. 279, 31277–31286. 10.1074/jbc.M400641200 15150278

[B66] Nishizawa H. Pryor-Koishi K. Kato T. Kowa H. Kurahashi H. Udagawa Y. (2007). Microarray analysis of differentially expressed fetal genes in placental tissue derived from early and late onset severe pre-eclampsia. Placenta 28, 487–497. 10.1016/j.placenta.2006.05.010 16860862

[B67] Nourizadeh-Lillabadi R. Seilo Torgersen J. Vestrheim O. Konig M. Alestrom P. Syed M. (2010). Early embryonic gene expression profiling of zebrafish prion protein (Prp2) morphants. PLoS ONE 5:e13573. 10.1371/journal.pone.0013573 21042590 PMC2962645

[B68] Nuvolone M. Kana V. Hutter G. Sakata D. Mortin-Toth S. M. Russo G.. (2013). SIRPalpha polymorphisms, but not the prion protein, control phagocytosis of apoptotic cells. J. Exp. Med. 210, 2539–2552. 10.1084/jem.20131274 24145514 PMC3832919

[B68a] O'Rourke K. I. Zhuang D. Truscott T. C. Yan H. Schneider D. A. (2011). Sparce PrP^Sc^ accumulation in the placentas of goats with naturally acquired scrapie. BMC Vet. Res. 7:7. 10.1186/1746-6148-7-7 21284878 PMC3041672

[B69] Ozkan Z. S. Simsek M. Ilhan F. Deveci D. Godekmerdan A. Sapmaz E. (2013). Plasma IL-17, IL-35, interferon-gamma, SOCS3 and TGF-beta levels in pregnant women with preeclampsia, and their relation with severity of disease. J. Matern. Fetal Neonatal Med. [Epub ahead of print]. 10.3109/14767058.2013.861415 24175856

[B70] Paisley D. Banks S. Selfridge J. McLennan N. F. Ritchie A. M. McEwan C.. (2004). Male infertility and DNA damage in Doppel knockout and prion protein/Doppel double-knockout mice. Am. J. Pathol. 164, 2279–2288. 10.1016/S0002-9440(10)63784-4 15161660 PMC1615753

[B71] Palmqvist L. Pineault N. Wasslavik C. Humphries R. K. (2007). Candidate genes for expansion and transformation of hematopoietic stem cells by NUP98-HOX fusion genes. PLoS ONE 2:e768. 10.1371/journal.pone.0000768 17712416 PMC1942085

[B72] Passet B. Halliez S. Beringue V. Laude H. Vilotte J. L. (2013). The prion protein family: looking outside the central nervous system. Prion 7, 127–130. 10.4161/pri.22851 23154632 PMC3609118

[B73] Passet B. Young R. Makhzami S. Vilotte M. Jaffrezic F. Halliez S.. (2012). Prion protein and Shadoo are involved in overlapping embryonic pathways and trophoblastic development. PLoS ONE 7:e41959. 10.1371/journal.pone.0041959 22860039 PMC3408428

[B74] Prusiner S. B. (1982). Novel proteinaceous infectious particles cause scrapie. Science 216, 136–144. 10.1126/science.6801762 6801762

[B75] Qin K. Coomaraswamy J. Mastrangelo P. Yang Y. Lugowski S. Petromilli C.. (2003). The PrP-like protein Doppel binds copper. J. Biol. Chem. 278, 8888–8896. 10.1074/jbc.M210875200 12482851

[B76] Reich A. Neretti N. Freiman R. N. Wessel G. M. (2012). Transcriptome variance in single oocytes within, and between, genotypes. Mol. Reprod. Dev. 79, 502–503. 10.1002/mrd.22061 22729953 PMC3418669

[B77] Resenberger U. K. Harmeier A. Woerner A. C. Goodman J. L. Muller V. Krishnan R.. (2011). The cellular prion protein mediates neurotoxic signalling of beta-sheet-rich conformers independent of prion replication. EMBO J. 30, 2057–2070. 10.1038/emboj.2011.86 21441896 PMC3098494

[B78] Robertson S. A. Prins J. R. Sharkey D. J. Moldenhauer L. M. (2013). Seminal fluid and the generation of regulatory T cells for embryo implantation. Am. J. Reprod. Immunol. 69, 315–330. 10.1111/aji.12107 23480148

[B79] Rossi D. Cozzio A. Flechsig E. Klein M. A. Rulicke T. Aguzzi A.. (2001). Onset of ataxia and Purkinje cell loss in PrP null mice inversely correlated with Dpl level in brain. EMBO J. 20, 694–702. 10.1093/emboj/20.4.694 11179214 PMC145426

[B80] Sawalha R. M. Brotherstone S. Conington J. Villanueva B. (2007a). Lambs with scrapie susceptible genotypes have higher postnatal survival. PLoS ONE 2:e1236. 10.1371/journal.pone.0001236 18043743 PMC2077931

[B81] Sawalha R. M. Brotherstone S. Man W. Y. Conington J. Bunger L. Simm G.. (2007b). Associations of polymorphisms of the ovine prion protein gene with growth, carcass, and computerized tomography traits in Scottish Blackface lambs. J. Anim. Sci. 85, 632–640. 10.2527/jas.2006-372 17040947

[B82] Schmitt-Ulms G. Ehsani S. Watts J. C. Westaway D. Wille H. (2009). Evolutionary descent of prion genes from the ZIP family of metal ion transporters. PLoS ONE 4:e7208. 10.1371/journal.pone.0007208 19784368 PMC2745754

[B83] Schneider B. Pietri M. Pradines E. Loubet D. Launay J. M. Kellermann O.. (2011). Understanding the neurospecificity of Prion protein signaling. Front. Biosci. (Landmark Ed). 16:169–186. 10.2741/3682 21196165

[B84] Screen M. Dean W. Cross J. C. Hemberger M. (2008). Cathepsin proteases have distinct roles in trophoblast function and vascular remodelling. Development 135, 3311–3320. 10.1242/dev.025627 18776147

[B85] Shaker O. G. Sadik N. A. (2013). Pathogenesis of preeclampsia: implications of apoptotic markers and oxidative stress. Hum. Exp. Toxicol. 32, 1170–1178. 10.1177/0960327112472998 23515498

[B86] Shi J. Feng H. Lee J. Ning Chen W. (2013). Comparative proteomics profile of lipid-cumulating oleaginous yeast: an iTRAQ-coupled 2-D LC-MS/MS analysis. PLoS ONE 8:e85532. 10.1371/journal.pone.0085532 24386479 PMC3873444

[B87] Shmerling D. Hegyi I. Fischer M. Blattler T. Brandner S. Gotz J.. (1998). Expression of amino-terminally truncated PrP in the mouse leading to ataxia and specific cerebellar lesions. Cell 93, 203–214. 10.1016/S0092-8674(00)81572-X 9568713

[B88] Sibai B. Dekker G. Kupferminc M. (2005). Pre-eclampsia. Lancet 365, 785–799. 10.1016/S0140-6736(05)17987-2 15733721

[B89] Singh A. Haldar S. Horback K. Tom C. Zhou L. Meyerson H.. (2013). Prion protein regulates iron transport by functioning as a ferrireductase. J. Alzheimers. Dis. 35, 541–552. 10.3233/JAD-130218 23478311 PMC5450724

[B90] Solis G. P. Radon Y. Sempou E. Jechow K. Stuermer C. A. Malaga-Trillo E. (2013). Conserved roles of the prion protein domains on subcellular localization and cell-cell adhesion. PLoS ONE 8:e70327. 10.1371/journal.pone.0070327 23936187 PMC3729945

[B91] Stanczuk G. A. McCoy M. J. Hutchinson I. V. Sibanda E. N. (2007). The genetic predisposition to produce high levels of TGF-beta1 impacts on the severity of eclampsia/pre-eclampsia. Acta Obstet. Gynecol. Scand. 86, 903–908. 10.1080/00016340701416945 17653872

[B92] Striebel J. F. Race B. Pathmajeyan M. Rangel A. Chesebro B. (2013). Lack of influence of prion protein gene expression on kainate-induced seizures in mice: studies using congenic, coisogenic and transgenic strains. Neuroscience 238, 11–18. 10.1016/j.neuroscience.2013.02.004 23415788 PMC3676307

[B93] Tanji K. Saeki K. Matsumoto Y. Takeda M. Hirasawa K. Doi K.. (1995). Analysis of PrPc mRNA by *in situ* hybridization in brain, placenta, uterus and testis of rats. Intervirology 38, 309–315. 8880380 10.1159/000150457

[B94] Taylor D. R. Hooper N. M. (2007). The low-density lipoprotein receptor-related protein 1 (LRP1) mediates the endocytosis of the cellular prion protein. Biochem. J. 402, 17–23. 10.1042/BJ20061736 17155929 PMC1783995

[B95] Thumdee P. Ponsuksili S. Murani E. Nganvongpanit K. Gehrig B. Tesfaye D.. (2007). Expression of the prion protein gene (*PRNP*) and cellular prion protein (PrPc) in cattle and sheep fetuses and maternal tissues during pregnancy. Gene Expr. 13, 283–297. 10.3727/000000006780666984 17605301 PMC6032460

[B96] Tian X. Anthony K. Neuberger T. Diaz F. J. (2014). Preconception zinc deficiency disrupts postimplantation fetal and placental development in mice. Biol. Reprod. 90, 83. 10.1095/biolreprod.113.113910 24599289 PMC4076385

[B97] Tremblay P. Bouzamondo-Bernstein E. Heinrich C. Prusiner S. B. Dearmond S. J. (2007). Developmental expression of PrP in the post-implantation embryo. Brain Res. 1139, 60–67. 10.1016/j.brainres.2006.12.055 17292334 PMC2706582

[B98] Turu M. Slevin M. Ethirajan P. Luque A. Elasbali A. Font A.. (2008). The normal cellular prion protein and its possible role in angiogenesis. Front. Biosci. 13, 6491–6500. 10.2741/3169 18508675

[B99] Uriu-Adams J. Y. Keen C. L. (2010). Zinc and reproduction: effects of zinc deficiency on prenatal and early postnatal development. Birth Defects Res. B Dev. Reprod. Toxicol. 89, 313–325. 10.1002/bdrb.20264 20803691

[B100] Wadley G. D. McConell G. K. Goodman C. A. Siebel A. L. Westcott K. T. Wlodek M. E. (2013). Growth restriction in the rat alters expression of metabolic genes during postnatal cardiac development in a sex-specific manner. Physiol. Genomics 45, 99–105. 10.1152/physiolgenomics.00095.2012 23232075

[B101] Wakefield S. L. Lane M. Mitchell M. (2011). Impaired mitochondrial function in the preimplantation embryo perturbs fetal and placental development in the mouse. Biol. Reprod. 84, 572–580. 10.1095/biolreprod.110.087262 21076083

[B102] Walter E. D. Stevens D. J. Spevacek A. R. Visconte M. P. Dei Rossi A. Millhauser G. L. (2009). Copper binding extrinsic to the octarepeat region in the prion protein. Curr. Protein Pept. Sci. 10, 529–535. 10.2174/138920309789352056 19538144 PMC2905140

[B103] Wang V. Chuang T. C. Hsu Y. D. Chou W. Y. Kao M. C. (2005). Nitric oxide induces prion protein via MEK and p38 MAPK signaling. Biochem. Biophys. Res. Commun. 333, 95–100. 10.1016/j.bbrc.2005.05.091 15936714

[B104] Watt N. T. Taylor D. R. Kerrigan T. L. Griffiths H. H. Rushworth J. V. Whitehouse I. J.. (2012). Prion protein facilitates uptake of zinc into neuronal cells. Nat. Commun. 3, 1134. 10.1038/ncomms2135 23072804 PMC3493655

[B105] Watts J. C. Huo H. Bai Y. Ehsani S. Jeon A. H. Shi T.. (2009). Interactome analyses identify ties of PrP and its mammalian paralogs to oligomannosidic N-glycans and endoplasmic reticulum-derived chaperones. PLoS Pathog. 5:e1000608. 10.1371/annotation/9eb11869-6acb-49b0-978e-abedc3cc545a 19798432 PMC2749441

[B106] Westergard L. Christensen H. M. Harris D. A. (2007). The cellular prion protein (PrP(C)): its physiological function and role in disease. Biochim. Biophys. Acta 1772, 629–644. 10.1016/j.bbadis.2007.02.011 17451912 PMC1986710

[B107] Wijshake T. Baker D. J. Van De Sluis B. (2014). Endonucleases: new tools to edit the mouse genome. Biochim. Biophys. Acta. [Epub ahead of print]. 10.1016/j.bbadis.2014.04.020 24794718

[B108] Wong B. S. Liu T. Paisley D. Li R. Pan T. Chen S. G.. (2001). Induction of HO-1 and NOS in doppel-expressing mice devoid of PrP: implications for doppel function. Mol. Cell. Neurosci. 17, 768–775. 10.1006/mcne.2001.0963 11312611

[B109] Wurm S. Wechselberger C. (2006). Prion protein modifies TGF-beta induced signal transduction. Biochem. Biophys. Res. Commun. 349, 525–532. 10.1016/j.bbrc.2006.08.074 16942751

[B110] Yamaguchi N. Sakaguchi S. Shigematsu K. Okimura N. Katamine S. (2004). Doppel-induced Purkinje cell death is stoichiometrically abrogated by prion protein. Biochem. Biophys. Res. Commun. 319, 1247–1252. 10.1016/j.bbrc.2004.05.115 15194501

[B111] Young R. Bouet S. Polyte J. Le Guillou S. Passet B. Vilotte M.. (2011). Expression of the prion-like protein Shadoo in the developing mouse embryo. Biochem. Biophys. Res. Commun. 416, 184–187. 10.1016/j.bbrc.2011.11.021 22093825

[B112] Young R. Passet B. Vilotte M. Cribiu E. P. Beringue V. Le Provost F.. (2009). The prion or the related Shadoo protein is required for early mouse embryogenesis. FEBS Lett. 583, 3296–3300. 10.1016/j.febslet.2009.09.027 19766638

[B113] Zadrozna M. Gawlik M. Nowak B. Marcinek A. Mrowiec H. Walas S.. (2009). Antioxidants activities and concentration of selenium, zinc and copper in preterm and IUGR human placentas. J. Trace Elem. Med. Biol. 23, 144–148. 10.1016/j.jtemb.2009.02.005 19398063

[B114] Zomosa-Signoret V. Arnaud J. D. Fontes P. Alvarez-Martinez M. T. Liautard J. P. (2008). Physiological role of the cellular prion protein. Vet. Res. 39, 9. 10.1051/vetres:2007048 18073096

